# Three-Dimensional-Enabled Surgical Planning for the Correction of Right Partial Anomalous Pulmonary Venous Return

**DOI:** 10.3390/jcm12020472

**Published:** 2023-01-06

**Authors:** Claudia Cattapan, Alvise Guariento, Olimpia Bifulco, Raphael Caraffa, Francesco Bertelli, Elena Reffo, Massimo Padalino, Giovanni Di Salvo, Vladimiro Vida

**Affiliations:** 1 Pediatric and Congenital Cardiac Surgery Unit, Department of Cardiac, Thoracic, Vascular Sciences and Public Health, University of Padua, 35128 Padua, Italy; 2 Pediatric Cardiology, Department of Pediatric and Maternal Medicine, University of Padua, 35128 Padua, Italy

**Keywords:** partial anomalous pulmonary venous return, 3D reconstruction, surgical planning, congenital heart disease, cardiac imaging

## Abstract

Objectives: The surgical technique for right partial anomalous pulmonary venous return (PAPVR) depends on the location of the anomalous pulmonary veins (PVs). With this in mind, we sought to evaluate the impact of 3D heart segmentation and reconstruction on preoperative surgical planning. Methods: A retrospective study was conducted on all patients who underwent PAPVR repair at our institution between January 2018 and October 2021; three-dimensional segmentations and reconstructions of all the heart anatomies were performed. A score (the PAPVR score) was established and calculated using two anatomical parameters (the distance between the most cranial anomalous PV and the superior rim of the sinus venosus defect/the sum of the latter and the distance between the PV and the azygos vein) to predict the type of correction. Results: A total of 30 patients were included in the study. The PAPVR score was found to be a good predictor of the type of surgery performed. A value < 0.68 was significantly associated with a Warden procedure (*p* < 0.001) versus single/double patch repair. Conclusions: Three-dimensional heart segmentations and reconstructions improve the quality of surgical planning in the case of PAPVR and allow for the introduction of a score that may facilitate surgical decisions on the type of repair required.

## 1. Introduction

Preoperative evaluation of congenital heart disease (CHD) still relies on 2D cross-sectional imaging. While providing useful information, this is sometimes inadequate for optimal patient-tailored surgical planning. In particular, the lack of a spatial representation can limit the correct choice of the most suitable corrective technique, especially when evaluating structures on different spatial planes. Consequently, the introduction of cardiac segmentation with 3D reconstruction of the heart may represent an interesting and useful preoperative diagnostic tool to help surgeons in the process of making the most appropriate therapeutic decision thanks to the possibility of obtaining additional measurements compared to 2D imaging [1,2,3].

Partial anomalous pulmonary venous return (PAPVR) is a rare congenital cardiac disease characterized by abnormal drainage of at least one pulmonary vein (PV) into the systemic venous circulation [4]. This is usually associated with a sinus venosus defect (SVD) in almost 90% of cases [5,6,7]. PAPVR correction can be performed through several procedures, which can be mainly divided into [8]: the single patch technique (baffling of the PVs to the left atrium through the SVD), the double patch technique (baffling and enlargement of the superior vena cava (SVC)) and the Warden technique, that is already extensively described in the literature [9,10]. It is recognized that the further the PVs are from the SVD, the more likely a Warden procedure should be performed due to the higher risk of obstruction to PVs’ baffling. Both types of correction are associated with potential risks for the patient. Single/double patch correction has the risk of creating an obstruction to the PVs’ baffling if the latter one is too long. The Warden procedure is associated with different potential risks based on the age of the patient: (1) in the adult population, a prosthetic conduit must be used to connect SVC to the right atrium and it requires anticoagulant therapy with warfarin, which causes an increased bleeding risk in the first postoperative months; (2) in younger patients, the length of the right atrial appendage may sometimes not be enough to ensure an unobstructed blood flow from SVC. There is currently no clear preoperative indication for a specific intervention, and the decision is still based on the surgeon’s experience [11].

Adequate preoperative planning is essential to present the patient and their family with all the possible strategies and inform them of the risks and consequences of the type of correction that will likely be performed.

The aim of this study was to determine a score that would guide the surgical decision on the procedure to perform a PAPVR repair using 3D reconstruction in order to improve preoperative planning and family/patient counselling.

## 2. Materials and Methods

**Figure 1:**
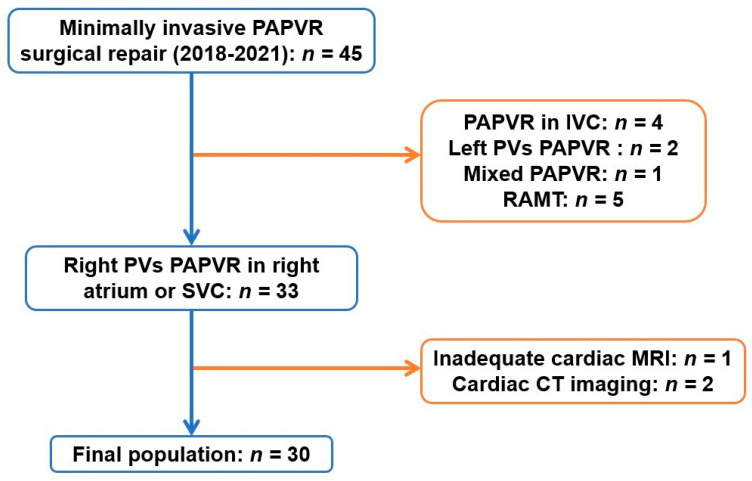
Flowchart showing the inclusion and exclusion criteria during the selection of the study population. CT: computed tomography; IVC: inferior vena cava; MRI: magnetic resonance imaging; PAPVR: partial anomalous pulmonary venous return; RAMT: right anterior minithoracotomy; SVC: superior vena cava.

This is a retrospective, observational, single-center study of all patients undergoing PAPVR repair between March 2018 and October 2021 at the University of Padua. Patients with PAPVR into the right atrium or SVC and with adequate pre-operative imaging to perform 3D reconstruction were included. To avoid the biases related to different imaging techniques, we decided to include only patients with a preoperative cardiac MRI. All left PAPVR and all drainages in the inferior vena cava (IVC) were excluded. Patients with cardiac MRI inadequate to achieve a proper 3D reconstruction (e.g., images with a slice thickness above 2 mm) and patients with only a cardiac CT scan were also excluded (Figure 1).

A minimally invasive surgical protocol is currently the preferred approach to correct PAPVR at our institution, using a right lateral (axillary) mini thoracotomy [12]. In the last three years, 3D reconstruction of the heart has been routinely performed preoperatively together with a chest wall reconstruction to plan the surgical access (Video S1).

A review of medical records was conducted in accordance with institutional guidelines for the retrospective review of medical records and the protection of patient confidentiality. Individual consent was not obtained from the patients enrolled in this study as the patients were not identified, and our institution’s ethics committee approved the submission of the data for publication (protocol number 4482/AO/18).

### 2.1. Image Acquisition Protocol and 3D Reconstruction

**Figure 2:**
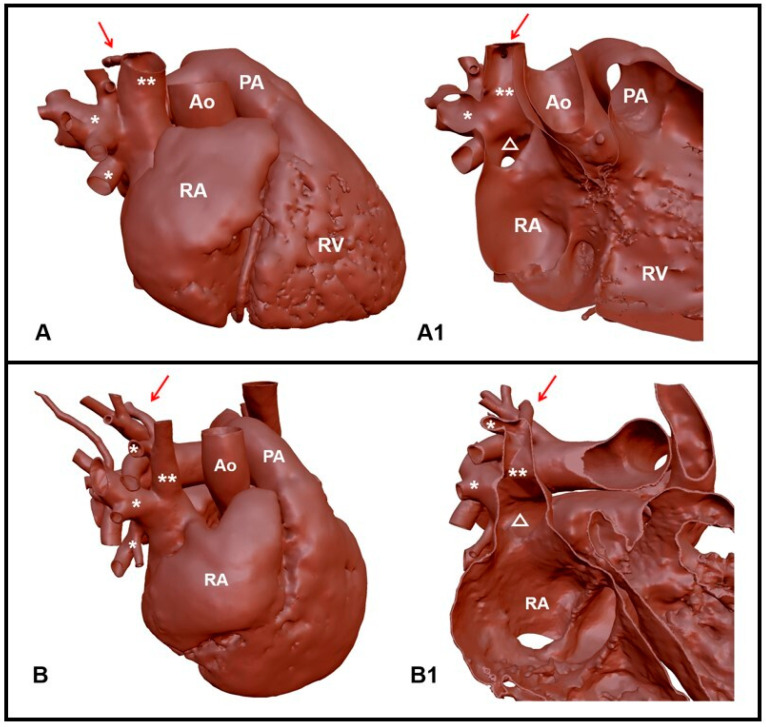
Three-dimensional reconstruction of two cases of partial anomalous pulmonary venous return in superior vena cava; the red arrow indicates the azygos vein. (**A**,**A1**) Case 1. (**A**) The anomalous pulmonary veins are displaced closer to the atrio-caval junction; (**A1**) coronal section; the sinus venosus defect is indicated by the white triangle. (**B**,**B1**) Case 2. (**B**) The most cranial anomalous pulmonary vein drains together with the azygos vein; (**B1**) coronal section; the sinus venosus defect is indicated by the white triangle. Ao: aorta; PA: pulmonary artery; RA: right atrium; RV: Right Ventricle; *: anomalous pulmonary veins; **: superior vena cava.

Standard protocols were used to obtain all of the images of the final population selected for the study. The specific procedure was designed according to the clinical diagnostic settings. Cardiac MRI was performed using a Philips Achieva 1.5 T scanner (Philips Medical Systems, Philips Healthcare, Amsterdam, The Netherlands). Images were acquired with a 5-channel phased-array cardiac coil. A 3D SSFP sequence, ECG, and free-breathing navigator gated scan were performed for the acquisition of the whole heart (TR/TE 3.1/1.56 ms, FOV 220 mm^3^, matrix 1923, and acquired voxel size 1.15 mm^3^). The thickness of the slices ranged from 1 mm to 2 mm. The acquisition field was extended from the supra-aortic trunks to the first portion of the abdominal aorta, just below its passage through the diaphragm. The 3D reconstruction process performed at our institution has been described previously [2] (Figure 2). The reconstruction phase usually takes about 2–3 h, depending on the quality of the cardiac MRI performed, while the printing phase generally takes 9 h.

### 2.2. Score Development and Image Analysis

The position of the anomalous PVs is generally considered among the most important parameters in preoperative surgical planning for the type of PAPVR correction required. For this reason, we decided to base our score on the distance between the superior rim of the most cranial anomalous PV and the SVD. However, this distance can vary considerably between patients at different stages of development. To obtain a score that can be applied to every patient, independent of BSA, we used the distance between the superior rim of the most cranial anomalous PV and the inferior rim of the azygos vein to normalize the results. These measurements were specifically performed on 3D reconstructed models of 2D cross-sectional imaging as the PVs and the SVD are in different spatial planes.

**Figure 3:**
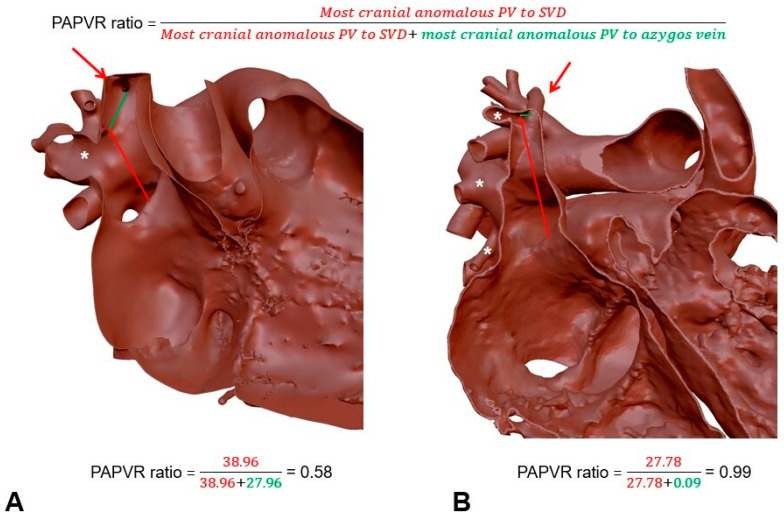
Determination of the PAPVR ratio. (**A**) The calculated formula suggests a double patch repair; and (**B**) the calculated formula suggests a Warden procedure. *: anomalous pulmonary veins; red arrow indicating the azygos vein.

The ratio obtained was defined as the predictive adjusted ratio for surgical planning in pulmonary anomalous venous connection repair ratio (or PAPVR ratio) and calculated from the formula: distance between the most cranial PV and the SVD (in mm) / [distance between the most cranial PV and the SVD (in mm) + distance between the cranial PV and the azygos vein (in mm)] (Figure 3).

### 2.3. Surgical Simulation

Starting from October 2021, we established a surgical simulation protocol with the aim to validate our score performing a perspective simulation. The surgeon first calculated the patient-specific PAPVR ratio and then performed the most suitable procedure on the model based on the predictive score calculated. These models were printed using a 3D printer (Form 2, Formlabs, Germany) and a specific elastic resin which made it possible to completely simulate the surgical procedure. If, based on the result obtained on the 3D-printed model, the surgeon considered the procedure adequate for the correction, he repeated it for the actual correction performed in the operating room.

### 2.4. Statistical Analysis

The surgical variables were expressed as the median and interquartile range (IQR) when continuous and as absolute and frequencies if categorical. A Student’s t-test was used for normally distributed variables, while a non-parametric Kruskal–Wallis signed-rank test was used for non-normal distributions. Fisher’s exact test or Pearson’s chi-square was used for nominal variables. Receiver operational characteristics (ROC) curves were plotted to identify the optimal score threshold for predicting a specific procedure. The statistical significance was set at a *p*-value < 0.05. STATA software version 15.0 (StataCorp. 2017. Stata Statistical Software: Release 15. College Station, TX: StataCorp LLC) was used for statistical analyses.

## 3. Results

A total of 30 patients were enrolled in the study (Flowchart, Figure 1). Patient characteristics, operational data, and outcomes according to the type of surgical procedure were recorded (Table S1).

### 3.1. PAPVR Score

A PAPVR score was calculated in each of the 30 patients (Table S2). The patients were grouped according to the surgical procedure performed, and the mean PAPVR score and standard deviation were determined (Table 1). The PAPVR score predicted the surgery performed. Indeed, as the score decreases, single and double patch techniques become the preferred procedure.

**Figure 4:**
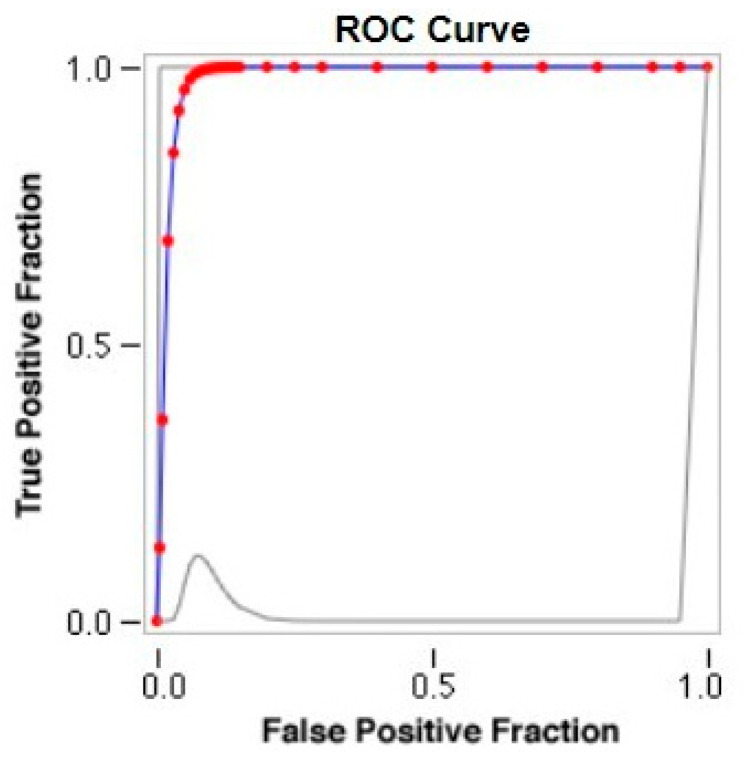
ROC curve of PAPVR ratio values in our population.

The PAPVR score was then used to obtain a cut-off value for the Warden procedure by comparing the score of patients undergoing this type of repair and those treated with single or double patch technique. A bimodal distribution of the values was found. Patients undergoing the Warden procedure were in the lower part of the curve, while the population of the single and double patch technique showed higher scores. The calculated ROC curve demonstrates that the maximum sensitivity (=0.93) and specificity (=1.0) in discriminating the two groups occurred for a PAPVR score value of 0.68 (Figure 4), which was identified as the cut-off point for proper surgical panning.

### 3.2. Surgical Simulation, PAPVR Score Validation, and Family Counselling

**Figure 5:**
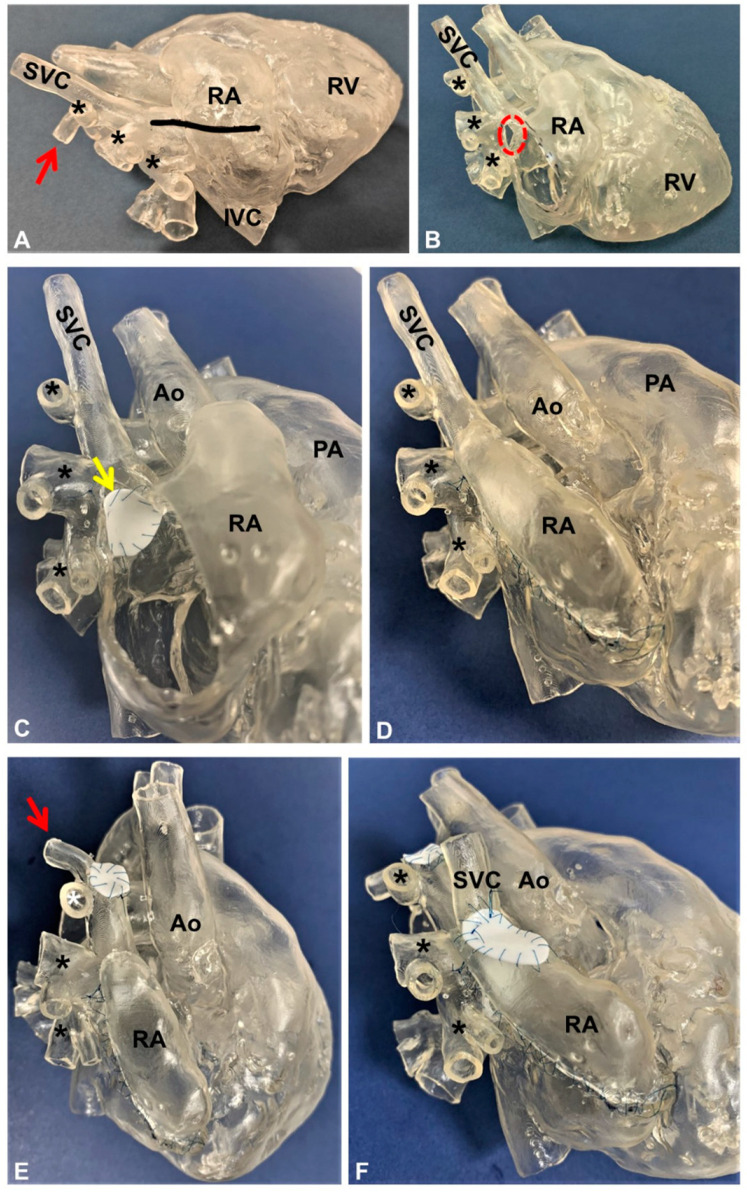
The 3D-printed model of a partial anomalous venous return (highlighted by black and white asterisks) corrected using the Warden technique. (**A**) An atriotomy is first performed as shown by the black line in the model; (**B**) the superior sinus venosus defect is now visible (highlighted by the red dotted circle); (**C**) A patch (yellow arrow) is sutured to baffle the pulmonary veins’ drainage through the sinus venosus defect and into the left atrium; (**D**) the right atriotomy is closed by direct suture; (**E**) the superior vena cava is resected just above the ostium of the most cranial pulmonary vein and a patch is used to close the proximal end of the vena cava itself; the azygos vein (red arrow) is also ligated (not shown in the image); (**F**) the distal end of the superior vena cava is anastomosed to the right atrium appendage and a patch is used to enlarge the anastomosis to avoid blood flow obstruction. Ao: aorta; IVC: inferior vena cava; PA: pulmonary artery; RA: right atrium; RV: right ventricle; SVC: superior vena cava.

**Figure 6:**
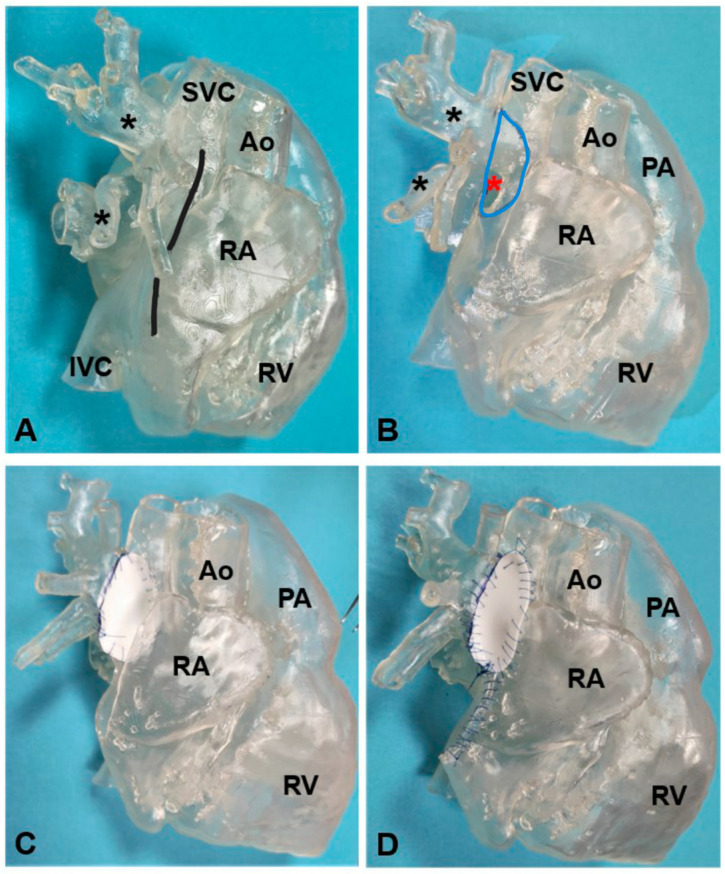
The 3D-printed model of a partial anomalous venous return (highlighted by black asterisks) corrected using the double patch technique. (**A**) An atriotomy is first performed as shown by the black line in the model; (**B**) the superior sinus venosus defect is now visible (highlighted by the red asterisk) through the superior half pf the right atriotomy (highlighted by the light blue line); (**C**) the first patch is sutured to baffle the pulmonary veins’ drainage through the sinus venosus defect and into the left atrium; (**D**) the second patch is used to enlarge the superior vena cava to avoid blood flow obstruction. Ao: aorta; IVC: inferior vena cava; PA: pulmonary artery; RA: right atrium; RV: right ventricle; SVC: superior vena cava.

A total of six 3D-printed hearts were prospectively evaluated from October 2021 to October 2022 to investigate the reliability of the cut-off we found. The calculated PAPVR ratio indicated that the Warden procedure could have been the most adequate technique for correction in three patients (Figure 5), who indeed obtained a PAPVR ratio > 0.68 (0.99, 0.82, and 0.88, respectively). The remaining three patients showed a ratio in favor of single/double patch correction (Figure 6). The specific surgical procedure suggested by the PAPVR ratio was then performed in the six models and it was considered adequate for correction in all the cases evaluated. When thereafter performed in the operating room, excellent results were obtained, and no flow gradients were observed in the postoperative transesophageal 2D echocardiography nor in the pre-discharge transthoracic one.

## 4. Discussion

Different surgical techniques can be used to re-establish a physiological connection in PAPVR, according to different parameters [13]. Therefore, a careful and complete evaluation of the patient’s specific anatomy is a fundamental requirement for accurate planning of the procedure to be performed in the operating room.

The surgical approach to correct PAPVR has changed at our institution over the years, moving from a classic median sternotomy to minimally invasive approaches [14,15]. These approaches allow better results in terms of postoperative pain and hospitalization, as well as better aesthetic outcomes [9,16,17]. A right lateral minithoracotomy is now the standard of care in PAPVR at our institution, providing a direct visualization of the right atrium and veins. Additionally, the use of peripheral cannulation and the placement of the superior venous cannula in the jugular vein provides an optimal view of the anomalous drainage and any possible associated SVD. This specific type of cannulation is particularly useful in PAPVR with abnormal PV(s) located very close to the azygos vein. In this specific setting, a careful preoperative evaluation is fundamental to direct the site of the incision.

In this study we sought to establish a score to guide surgical decisions in PAPVR repair. The possibility to predict the type of intervention before entering the operating room would help us to individuate the intercostal space in which the incision is performed to obtain the best exposure of the desired structures (e.g., entering a more cranial intercostal space is preferred in the case of a Warden procedure). The score was based on the idea that the more cranially the anomalous PVs are, the more likely an obstruction to blood flow may result from the tunnel created using a patch. Thus, the Warden technique would be the preferred procedure in the case of an elevated distance between the PVs and the SVD. Since this measure differs according to the dimensions of the patients, we decided to obtain a ratio that would be universally applicable. A decision was made to normalize our values using the position of the azygos vein, and in particular its distance from the SVD.

Based on our experience in image analysis, we performed these measurements on our 3D reconstructed models. Indeed, previous studies have shown the impossibility to accurately measure the distances required for the PAPVR ratio using 2D imaging. Three-dimensional reconstruction is the only modality to obtain accurate measurements of the distance between structures located on completely different spatial planes.

Our PAPVR ratio showed a positive correlation with the technique performed. In addition to this, a cut-off between the single/double patch technique and the Warden procedure was identified. In particular a ratio above 0.68 positively predicts the latter procedure.

This new score may represent an innovative tool to improve preoperative planning in PAPVR allowing a more accurate definition of the surgical procedure to be performed in the operating room. In this way, the surgeon can possibly predict not only the type of intervention but can also design the required patches. Counselling with parents would also benefit from this score through informing the family of all of the possible scenarios (such as the need for anticoagulation in the case of prosthesis implantation for the Warden procedure) [18]. This can undoubtedly improve dialogue, making families more aware about the child’s condition and strengthening the relationship with the clinician.

The possibility to print 3D models to simulate the procedure represents a further improvement in surgical planning using 3D reconstruction [19,20]. As we have shown, patient-specific 3D-printed models can be obtained in a relatively short time and can be reprinted as many times as desired. This could possibly help the surgeon to identify the most appropriate surgical technique and limit unexpected events during the intervention. Finally, 3D-printed models could represent an innovative tool for surgical education by enabling the training of young surgeons in a totally safe environment [21,22,23].

Based on our positive experience with preoperative counselling using 3D-printed patient-specific hearts [24], starting from 2018, we have routinely used the 3D-printed model (in solid resin) when obtaining informed consent in order to explain to the patient and their family the pathology and the procedure that will be likely performed.

### Limitations

The main limitations of this study are the retrospective design and the limited sample size. Although our previous case series represent one of the most numerous series of minimally invasive PAPVR correction, only patients with a specific MRI protocol could be evaluated for the impact of 3D reconstruction; however cardiac CT scan can also be used to obtain 3D-reconstructed hearts. Furthermore, to eliminate all potential confounders, only a specific type of PAPVR was included, causing a significant reduction in the number of the population.

## 5. Conclusions

Three-dimensional heart segmentation and reconstruction improve the quality of PAPVR surgical planning. A PAPVR score may facilitate the surgeon’s decision regarding the type of correction required. Further studies are needed to validate this parameter.

## Figures and Tables

**Table 1:** Mean PAPVR ratio value according to the surgical procedure performed.

Type of Correction	Mean	Standard Deviation	*p*-Value
Single patch technique	0.51	0.08	<0.001
Double patch technique	0.62	0.09	<0.001
Warden procedure	0.85	0.10	<0.001

## Data Availability

The data presented in this study are available on request from the corresponding author. The data are not publicly available due to ethical restrictions.

## Supplementary Materials

The following supporting information can be downloaded at: https://www.mdpi.com/article/10.3390/jcm12020472/s1, Table S1: Characteristics, operative data, and patient results; Table S2: Patients’ specific ratio value; Video S1: Right lateral minithoracotomy approach to partial anomalous pulmonary venous return.
