# Supplementary material for: Three-Dimensional-Enabled Surgical Planning for the Correction of Right Partial Anomalous Pulmonary Venous Return
Source: J Clin Med. 2023 Jan 6;12(2):472. doi: 10.3390/jcm12020472 (PMC9863474; doi:10.3390/jcm12020472)
Supplement: Supplementary file 1 [file jcm-12-00472-s001.zip › Supplementary material.pdf]

Table S1. Characteristics, operative data, and patient results (n=30)

Variables	Single/Double Patch technique (n=15)	Warden procedure (n=15)	p-value
**Characteristics**			
Males*	11 (73)	9 (60)	0.44
Associated ASD*	14 (93)	13 (87)	0.55
Age at surgery (months)**	86 (50-125)	116 (44-164)	0.46
Weight at surgery (kilograms)**	21 (15.7-33)	30.8 (14.2-52)	0.51
**Operative data**			
Peripheral cannulation*	14 (93)	13 (87)	0.55
CPB time (minutes)**	57 (44-67)	117 (77-132)	<0.001
CC time (minutes)**	42 (31-50)	60 (42-88)	0.005
**Outcomes**			
ICU (days)**	1 (1-1)	1 (1-2)	0.17
Post-operative complications	0 (0)	3 (20)	0.07
• Post-pericardiotomy syndrome	0	2	
• Temporary atrial fibrillation	0	1	

Hospital length of stay (days)**	5 (4-6)	5 (3-8)	0.22

*data presented as number of complications and percentage (%); **data presented as median and interquartile range. ASD: atrial septal defect; VSD: ventricular septal defect; CPB: Cardio-pulmonary bypass; CC: Cross-clamp; ICU: Intensive care unit.

Table S2. Patients' specific ratio value.

Number	Gender (M, F)	Age (months)	PVs to ASD (mm)	AV to PVs (mm)	Ratio	Surgical procedure performed
1	F	164	39.08	0.17	0.99	Warden procedure
2	F	44	26.18	3.61	0.88	Warden procedure
3	M	130	38.45	13.43	0.74	Warden procedure
4	M	125	35.99	21.49	0.63	Double patch
5	M	59	27.69	12.84	0.68	Double patch
6	M	50	17.41	12.27	0.59	Double patch
7	F	37	31.02	7.20	0.81	Warden procedure
8	F	139	36.06	15.93	0.69	Warden procedure
9	M	116	40.16	10.96	0.79	Warden procedure
10	F	26	25.23	3.02	0.89	Warden procedure
11	M	82	24.74	12.69	0.66	Single patch
12	F	48	24.91	10.25	0.71	Warden procedure
13	M	32	13.67	12.17	0.53	Single patch
14	F	803	48.24	0.23	0.99	Warden procedure

15	M	114	33.93	22.29	0.60	Double patch
16	M	65	24.90	21.75	0.53	Single patch
17	M	558	47.32	7.14	0.87	Warden procedure
18	F	232	44.57	8.63	0.84	Warden procedure
19	F	152	26.83	38.43	0.41	Single patch
20	M	86	36.16	9.63	0.79	Double patch
21	M	70	28.63	5.88	0.83	Warden procedure
22	M	18	12.81	11.88	0.52	Single patch
23	F	100	23.60	27.97	0.46	Double patch
24	M	154	46.52	8.97	0.84	Warden procedure
25	M	453	38.96	27.76	0.58	Double patch
26	M	43	14.82	17.33	0.46	Single patch
27	M	106	50.02	11.20	0.82	Warden procedure
28	M	94	24.71	24.72	0.50	Single patch
29	M	454	26.84	14.68	0.65	Double patch
30	F	44	27.78	0.09	0.99	Warden procedure

ASD: atrial septal defect; AV: azygos vein; PVs: pulmonary veins.
